# [2,6-Bis(*p*-tol­ylimino­meth­yl)pyridine-κ^3^ *N*,*N*′,*N*′′]dichloridocopper(II)

**DOI:** 10.1107/S1600536810037025

**Published:** 2010-09-25

**Authors:** Xiao-Ping Li, Jian-She Zhao, Seik Weng Ng

**Affiliations:** a Department of Chemistry, Shaanxi Key Laboratory for Physico-Inorganic Chemistry, Northwest University, Xi’an 710069, People’s Republic of China; b Department of Chemistry, University of Malaya, 50603 Kuala Lumpur, Malaysia

## Abstract

The title compound, [CuCl_2_(C_21_H_19_N_3_)], lies on a twofold rotation axis that passes through the N_pyrid­yl_—Cu bond; this symmetry element relates one half of the organic ligand to the other as well as one Cl ligand to the other. The three N atoms span the axial–equatorial–axial sites of the trigonal-bipyramidal coordination polyhedron; the geometry of the Cu^II^ atom is 31% distorted from trigonal-bipyramidal (towards square-pyramidal along the Berry pseudorotation pathway).

## Related literature

For a chromium chloride adduct with a similar ligand, see: Li *et al.* (2010 ▶).
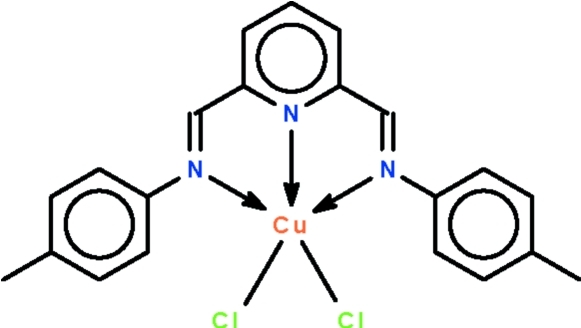


## Experimental

### 

#### Crystal data

[CuCl_2_(C_21_H_19_N_3_)]
*M* *_r_* = 447.83
Orthorhombic, 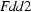

*a* = 11.5220 (13) Å
*b* = 35.522 (4) Å
*c* = 9.327 (1) Å
*V* = 3817.4 (7) Å^3^
*Z* = 8
Mo *K*α radiation
μ = 1.44 mm^−1^
*T* = 100 K
0.36 × 0.12 × 0.02 mm

#### Data collection

Bruker SMART APEX diffractometer
Absorption correction: multi-scan (*SADABS*; Sheldrick, 1996 ▶) *T*_min_ = 0.626, *T*_max_ = 0.972
8753 measured reflections
2190 independent reflections
2023 reflections with *I* > 2σ(*I*)
*R*_int_ = 0.050

#### Refinement

*R*[*F* ^2^ > 2σ(*F* ^2^)] = 0.030
*wR*(*F* ^2^) = 0.070
*S* = 1.04
2190 reflections
125 parameters
1 restraint
H-atom parameters constrained
Δρ_max_ = 0.29 e Å^−3^
Δρ_min_ = −0.30 e Å^−3^
Absolute structure: Flack (1983 ▶), 858 Friedel pairs
Flack parameter: 0.014 (14)

### 

Data collection: *APEX2* (Bruker, 2009 ▶); cell refinement: *SAINT* (Bruker, 2009 ▶); data reduction: *SAINT*; program(s) used to solve structure: *SHELXS97* (Sheldrick, 2008 ▶); program(s) used to refine structure: *SHELXL97* (Sheldrick, 2008 ▶); molecular graphics: *X-SEED* (Barbour, 2001 ▶); software used to prepare material for publication: *publCIF* (Westrip, 2010 ▶).

## Supplementary Material

Crystal structure: contains datablocks global, I. DOI: 10.1107/S1600536810037025/xu5030sup1.cif

Structure factors: contains datablocks I. DOI: 10.1107/S1600536810037025/xu5030Isup2.hkl

Additional supplementary materials:  crystallographic information; 3D view; checkCIF report

## Figures and Tables

**Table 1:** Selected bond lengths (Å)

Cu1—N1	1.968 (3)
Cu1—N2	2.101 (2)
Cu1—Cl1	2.3187 (7)

**Fig. 1.**
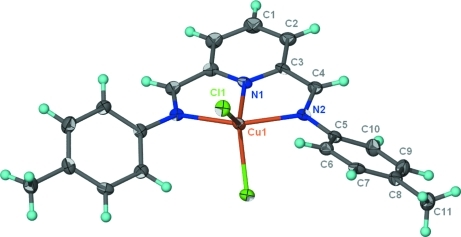
Thermal ellipsoid plot (Barbour, 2001) of CuCl2(C21H19N3) at the 70% probability level; hydrogen atoms are drawn as spheres of arbitrary radius.

## supplementary crystallographic information

### Comment

A recent study reported the chromium(III) chloride adduct of 2,6-bis(*p*-bromphenylimino)pyridine; the *N*-heterocycle chelates to the metal atom in a terdentate manner (Li *et al.*, 2010). The copper dichlroide adduct of 2,6-bis(*p*-tolylimino)pyridine adopts a similar structure. The CuCl_2_(C_21_H_19_N_3_) molecule (Scheme I, Fig. 1) lies on a twofold rotation axis that passes through the N_pyridyl_—Cu bond; this symmetry element relates one half of the organic ligand to the other. The three N atoms span the axial–equatorial-axial sites of the trigonal bipyramidal coordination polyhedron; the geometry of Cu is 31% distorted along the Berry pseudorotation pathway.

### Experimental

2,6-Bis(*p*-tolylimino)pyridine (0.016 g, 0.05 mmol), and copper chloride dihydrate (0.01 g, 0.05 mmol) along with five drops of 1 *M* hydrochloric acid were dissolved in ethanol (10 ml). The mixture was heated in a Teflon-lined, stainless-steel Parr bomb at 363 K for 120 h. The bomb was cooled at 5 K per hour. Deep orange crystals were isolated.

### Refinement

Carbon-bound H-atoms were placed in calculated positions (C—H 0.95 to 0.98 Å) and were included in the refinement in the riding model approximation, with *U*(H) set to 1.2 to 1.5*U*(C).

### Crystal data

**Table:**

[CuCl_2_(C_21_H_19_N_3_)]	*F*(000) = 1832
*M**_r_* = 447.83	*D*_x_ = 1.558 Mg m^−^^3^
Orthorhombic, *F**d**d*2	Mo *K*α radiation, λ = 0.71073 Å
Hall symbol: F 2 -2d	Cell parameters from 2394 reflections
*a* = 11.5220 (13) Å	θ = 2.3–26.1°
*b* = 35.522 (4) Å	µ = 1.44 mm^−^^1^
*c* = 9.327 (1) Å	*T* = 100 K
*V* = 3817.4 (7) Å^3^	Prism, orange
*Z* = 8	0.36 × 0.12 × 0.02 mm

### Data collection

**Table:**

Bruker SMART APEX diffractometer	2190 independent reflections
Radiation source: fine-focus sealed tube	2023 reflections with *I* > 2σ(*I*)
graphite	*R*_int_ = 0.050
ω scans	θ_max_ = 27.5°, θ_min_ = 2.3°
Absorption correction: multi-scan (*SADABS*; Sheldrick, 1996)	*h* = −13→14
*T*_min_ = 0.626, *T*_max_ = 0.972	*k* = −46→46
8753 measured reflections	*l* = −12→12

### Refinement

**Table:**

Refinement on *F*^2^	Secondary atom site location: difference Fourier map
Least-squares matrix: full	Hydrogen site location: inferred from neighbouring sites
*R*[*F*^2^ > 2σ(*F*^2^)] = 0.030	H-atom parameters constrained
*wR*(*F*^2^) = 0.070	*w* = 1/[σ^2^(*F*_o_^2^) + (0.0349*P*)^2^] where *P* = (*F*_o_^2^ + 2*F*_c_^2^)/3
*S* = 1.04	(Δ/σ)_max_ = 0.001
2190 reflections	Δρ_max_ = 0.29 e Å^−^^3^
125 parameters	Δρ_min_ = −0.30 e Å^−^^3^
1 restraint	Absolute structure: Flack (1983), 858 Friedel pairs
Primary atom site location: structure-invariant direct methods	Flack parameter: 0.014 (14)

### Fractional atomic coordinates and isotropic or equivalent isotropic displacement parameters (Å^2^)

**Table:**

	*x*	*y*	*z*	*U*_iso_*/*U*_eq_	
Cu1	1.0000	0.5000	0.50991 (4)	0.01349 (12)	
Cl1	0.90550 (6)	0.543930 (17)	0.36785 (8)	0.01783 (15)	
N1	1.0000	0.5000	0.7209 (3)	0.0136 (7)	
N2	0.8670 (2)	0.46146 (6)	0.5570 (2)	0.0137 (5)	
C1	1.0000	0.5000	1.0139 (7)	0.0233 (8)	
H1	1.0000	0.5000	1.1158	0.028*	
C2	0.9203 (3)	0.47824 (8)	0.9392 (3)	0.0197 (6)	
H2	0.8648	0.4634	0.9889	0.024*	
C3	0.9231 (2)	0.47860 (7)	0.7896 (3)	0.0148 (6)	
C4	0.8502 (3)	0.45700 (8)	0.6925 (3)	0.0159 (6)	
H4	0.7924	0.4403	0.7276	0.019*	
C5	0.8043 (2)	0.43861 (7)	0.4590 (3)	0.0146 (5)	
C6	0.7745 (2)	0.45364 (7)	0.3259 (3)	0.0162 (6)	
H6	0.7996	0.4782	0.3003	0.019*	
C7	0.7085 (2)	0.43273 (7)	0.2314 (3)	0.0155 (6)	
H7	0.6854	0.4436	0.1429	0.019*	
C8	0.6752 (2)	0.39602 (7)	0.2636 (3)	0.0184 (6)	
C9	0.7099 (3)	0.38078 (8)	0.3943 (3)	0.0220 (6)	
H9	0.6893	0.3556	0.4171	0.026*	
C10	0.7736 (2)	0.40150 (8)	0.4910 (3)	0.0195 (6)	
H10	0.7966	0.3906	0.5796	0.023*	
C11	0.6078 (3)	0.37314 (8)	0.1571 (3)	0.0233 (6)	
H11A	0.5523	0.3571	0.2079	0.035*	
H11B	0.6613	0.3574	0.1017	0.035*	
H11C	0.5659	0.3900	0.0921	0.035*	

### Atomic displacement parameters (Å^2^)

**Table:**

	*U*^11^	*U*^22^	*U*^33^	*U*^12^	*U*^13^	*U*^23^
Cu1	0.0124 (2)	0.0191 (2)	0.0089 (2)	−0.00124 (19)	0.000	0.000
Cl1	0.0175 (3)	0.0185 (3)	0.0175 (3)	0.0010 (3)	−0.0038 (3)	0.0027 (3)
N1	0.0098 (15)	0.0178 (15)	0.0131 (17)	0.0039 (13)	0.000	0.000
N2	0.0135 (12)	0.0152 (11)	0.0124 (11)	0.0023 (9)	0.0014 (9)	0.0001 (8)
C1	0.031 (2)	0.0271 (18)	0.0118 (18)	0.000 (2)	0.000	0.000
C2	0.0255 (16)	0.0207 (15)	0.0129 (14)	0.0006 (11)	0.0038 (11)	−0.0004 (11)
C3	0.0139 (14)	0.0179 (13)	0.0124 (16)	0.0031 (10)	0.0043 (11)	0.0007 (10)
C4	0.0180 (15)	0.0165 (13)	0.0133 (14)	0.0010 (11)	0.0023 (11)	0.0002 (11)
C5	0.0134 (13)	0.0177 (13)	0.0126 (13)	−0.0007 (11)	0.0018 (11)	−0.0017 (10)
C6	0.0166 (13)	0.0152 (12)	0.0168 (14)	0.0013 (11)	0.0016 (11)	0.0001 (11)
C7	0.0158 (13)	0.0211 (13)	0.0095 (14)	0.0048 (11)	0.0007 (10)	−0.0007 (10)
C8	0.0153 (13)	0.0215 (13)	0.0183 (14)	−0.0026 (10)	0.0005 (14)	−0.0032 (13)
C9	0.0271 (15)	0.0187 (14)	0.0201 (16)	−0.0057 (11)	0.0014 (13)	0.0012 (11)
C10	0.0212 (15)	0.0196 (13)	0.0177 (16)	−0.0026 (10)	−0.0009 (12)	0.0043 (12)
C11	0.0252 (16)	0.0243 (15)	0.0203 (16)	−0.0069 (13)	−0.0020 (12)	−0.0011 (12)

### Geometric parameters (Å, °)

**Table:**

Cu1—N1	1.968 (3)	C4—H4	0.9500
Cu1—N2^i^	2.101 (2)	C5—C6	1.394 (4)
Cu1—N2	2.101 (2)	C5—C10	1.397 (4)
Cu1—Cl1	2.3187 (7)	C6—C7	1.381 (4)
Cu1—Cl1^i^	2.3187 (7)	C6—H6	0.9500
N1—C3^i^	1.332 (3)	C7—C8	1.392 (4)
N1—C3	1.332 (3)	C7—H7	0.9500
N2—C4	1.288 (3)	C8—C9	1.392 (4)
N2—C5	1.421 (4)	C8—C11	1.500 (4)
C1—C2^i^	1.388 (5)	C9—C10	1.377 (4)
C1—C2	1.388 (5)	C9—H9	0.9500
C1—H1	0.9500	C10—H10	0.9500
C2—C3	1.396 (3)	C11—H11A	0.9800
C2—H2	0.9500	C11—H11B	0.9800
C3—C4	1.454 (4)	C11—H11C	0.9800
			
N1—Cu1—N2^i^	77.92 (7)	N2—C4—H4	121.3
N1—Cu1—N2	77.92 (7)	C3—C4—H4	121.3
N2^i^—Cu1—N2	155.85 (13)	C6—C5—C10	119.3 (3)
N1—Cu1—Cl1	124.85 (2)	C6—C5—N2	118.7 (2)
N2^i^—Cu1—Cl1	91.35 (6)	C10—C5—N2	122.0 (2)
N2—Cu1—Cl1	102.45 (7)	C7—C6—C5	119.8 (2)
N1—Cu1—Cl1^i^	124.85 (2)	C7—C6—H6	120.1
N2^i^—Cu1—Cl1^i^	102.45 (7)	C5—C6—H6	120.1
N2—Cu1—Cl1^i^	91.35 (6)	C6—C7—C8	121.2 (3)
Cl1—Cu1—Cl1^i^	110.30 (4)	C6—C7—H7	119.4
C3^i^—N1—C3	122.4 (3)	C8—C7—H7	119.4
C3^i^—N1—Cu1	118.78 (17)	C7—C8—C9	118.3 (3)
C3—N1—Cu1	118.78 (17)	C7—C8—C11	120.5 (3)
C4—N2—C5	119.0 (2)	C9—C8—C11	121.2 (2)
C4—N2—Cu1	113.3 (2)	C10—C9—C8	121.3 (3)
C5—N2—Cu1	127.49 (18)	C10—C9—H9	119.4
C2^i^—C1—C2	119.7 (5)	C8—C9—H9	119.4
C2^i^—C1—H1	120.1	C9—C10—C5	119.9 (3)
C2—C1—H1	120.1	C9—C10—H10	120.0
C1—C2—C3	118.8 (4)	C5—C10—H10	120.0
C1—C2—H2	120.6	C8—C11—H11A	109.5
C3—C2—H2	120.6	C8—C11—H11B	109.5
N1—C3—C2	120.2 (3)	H11A—C11—H11B	109.5
N1—C3—C4	112.7 (2)	C8—C11—H11C	109.5
C2—C3—C4	127.2 (3)	H11A—C11—H11C	109.5
N2—C4—C3	117.3 (3)	H11B—C11—H11C	109.5
			
N2^i^—Cu1—N1—C3^i^	−1.14 (14)	C1—C2—C3—N1	−1.1 (4)
N2—Cu1—N1—C3^i^	178.86 (14)	C1—C2—C3—C4	177.8 (2)
Cl1—Cu1—N1—C3^i^	−84.27 (13)	C5—N2—C4—C3	174.8 (2)
Cl1^i^—Cu1—N1—C3^i^	95.73 (13)	Cu1—N2—C4—C3	0.1 (3)
N2^i^—Cu1—N1—C3	178.86 (14)	N1—C3—C4—N2	−1.0 (4)
N2—Cu1—N1—C3	−1.14 (14)	C2—C3—C4—N2	−180.0 (3)
Cl1—Cu1—N1—C3	95.73 (13)	C4—N2—C5—C6	148.0 (3)
Cl1^i^—Cu1—N1—C3	−84.27 (13)	Cu1—N2—C5—C6	−38.1 (3)
N1—Cu1—N2—C4	0.5 (2)	C4—N2—C5—C10	−33.1 (4)
N2^i^—Cu1—N2—C4	0.5 (2)	Cu1—N2—C5—C10	140.8 (2)
Cl1—Cu1—N2—C4	−122.9 (2)	C10—C5—C6—C7	4.4 (4)
Cl1^i^—Cu1—N2—C4	126.0 (2)	N2—C5—C6—C7	−176.7 (2)
N1—Cu1—N2—C5	−173.7 (2)	C5—C6—C7—C8	−3.0 (4)
N2^i^—Cu1—N2—C5	−173.7 (2)	C6—C7—C8—C9	0.2 (4)
Cl1—Cu1—N2—C5	62.9 (2)	C6—C7—C8—C11	−177.4 (3)
Cl1^i^—Cu1—N2—C5	−48.3 (2)	C7—C8—C9—C10	1.3 (4)
C2^i^—C1—C2—C3	0.5 (2)	C11—C8—C9—C10	178.8 (3)
C3^i^—N1—C3—C2	0.6 (2)	C8—C9—C10—C5	0.1 (4)
Cu1—N1—C3—C2	−179.4 (2)	C6—C5—C10—C9	−3.0 (4)
C3^i^—N1—C3—C4	−178.5 (2)	N2—C5—C10—C9	178.2 (3)
Cu1—N1—C3—C4	1.5 (2)		

Symmetry codes: (i) −*x*+2, −*y*+1, *z*.
